# Gadobutrol versus gadofosveset-trisodium in MR venography of the lower extremities

**DOI:** 10.1007/s00330-017-4902-0

**Published:** 2017-07-03

**Authors:** Carsten W. K. P. Arnoldussen, Yeelai Lam, Nobutake Ito, Bjorn Winkens, M. Eline Kooi, Cees H. A. Wittens, Joachim E. Wildberger

**Affiliations:** 1 Department of Radiology, Maastricht University Medical Center, P.O. Box 5800, P.Debyelaan 25, 6202 AZ Maastricht, The Netherlands; 2 Department of Radiology, VieCuri Medical Center, Venlo, The Netherlands; 3 Department of Vascular Surgery, Maastricht University Medical Center, Maastricht, The Netherlands; 4 Department of Diagnostic Radiology, National Hospital Organization Tokyo Medical Center, Tokyo, Japan; 5 Department of Diagnostic Radiology, Keio University Hospital, Tokyo, Japan; 6 Department of Methodology and Statistics, Maastricht University, Maastricht, The Netherlands; 7 Maastricht University Medical Center, CARIM – School for Cardiovascular Diseases, Maastricht, The Netherlands; 8 Department of Vascular Surgery, University Hospital RWTH Aachen, Aachen, Germany

**Keywords:** MRI, MR venography, Venous, Chronic venous disease, Chronic venous obstruction

## Abstract

**Objectives:**

MR venography (MRV) protocols have used bloodpool contrast agents and long scan sequences to identify patients suitable for treatment and preoperatively. However, variable availability of bloodpool contrast agents, high costs and a need to shorten acquisition times for routine MR protocols hamper everyday practice.

**Materials:**

20 patients (11 men; mean age 54 ± 11.8 years; body mass index 23.6 ± 2.5) were enrolled in this prospective study. An intra-individual comparison of image quality, interpretation and findings for two different contrast agents (regular gadolinium contrast agent gadobutrol vs. bloodpool contrast agent gadofosveset-trisodium) and two different scan protocols (long acquisition time protocol using a high-resolution fast field echo (FFE) sequence vs. short acquisition time protocol using an ultra-fast gradient echo (GE) sequence) were performed.

**Results:**

Image quality (average of 4.94 vs. 4.92 on a five-point scale), interpretation and contrast-to-noise ratio (44 vs. 45) were equal for both contrast agents. Image findings showed no statistical significant differences between the MR protocols or contrast agents (overall *p* = 0.328).

**Conclusions:**

For high-resolution MRV, it is possible to replace gadofosveset-trisodium with gadobutrol. Furthermore, an ultra-fast GE sequence for MRV might considerably shorten acquisition time, without loss of image quality or diagnostic yield.

***Key Points*:**

• *High-quality MRV can be performed with a regular gadolinium-based contrast agent*.

• *Ultra-fast GRE vs. HR-FFE MRV: equally suitable for evaluation of venous obstruction*.

• *Regular gadolinium-based contrast agent can supersede a bloodpool contrast agent for MRV*.

• *Equal confidence for gadobutrol vs gadofosveset-trisodium in MRV*.

• *MRV accessible for routine daily practice*.

## Introduction

With the introduction and success of minimally invasive treatment options for chronic venous obstructive disease, imaging of abdomino-pelvic and lower extremity veins is receiving increased attention [1]. Chronic venous obstructive disease is defined as post-thrombotic obstructive disease of the deep veins, in particular, at the level of the iliocaval confluence and or (proximal) femoral veins, which results in impaired deep vein outflow. An addition to the above definition is the group of chronic venous obstructive lesions which are not related to deep vein thrombosis, called non-thrombotic iliac vein lesions (NIVLs). [2]. Identification of such deep vein disease can be performed with duplex ultrasound, computed tomography venography (CTV) or magnetic resonance venography (MRV). In particular, above the groin, MRV is more suitable to accurately identify the location of deep vein obstruction and chronic sequela of previous deep vein thrombosis events as well as provide an anatomic overview in the pre-interventional work-up [3–7].

Several studies have shown that the use of blood pool agents is favourable, due to the creation of a long steady-state imaging window for the high-resolution acquisition of the entire deep venous system in the lower extremities, allowing for detailed depiction of the (intra)luminal changes [8–11]. However, in the clinical arena, we are currently facing a three-fold problem: First, bloodpool contrast agents are expensive. Secondly, the most commonly used bloodpool agent for vascular imaging, Ablavar, is no longer commercially available in Europe. Thirdly, the clinical acceptance of these MR protocols is limited due to the relatively long acquisition time which easily exceeds 25 min [6].

An alternative technique to acquire large-volume, high-resolution 3D images is a high-resolution 3D T1-weighted volume interpolated gradient echo (GE) sequence with fat suppression (ultrafast GE) [10, 11]. This sequence has the potential to greatly reduce acquisition time for the required (large) volume. Acquisition time of less than 20 min might form the basis for the broad use of conventional extracellular gadolinium contrast agents [12–14].

Our goal for this study was to provide a clinical alternative to gadofosveset-trisodium by using a globally available extracellular gadolinium-based contrast agent instead. Secondly, we optimized a shorter yet robust acquisition protocol for lower extremity MRV to be used in daily clinical practise.

## Material and methods

### Patients

During an 8-month period, 129 consecutive patients seen at our dedicated venous out-patient clinic with clinical signs of chronic deep vein obstruction were invited to participate in this prospective study. Clinical signs included a CEAP classification of 4 or more, a Villalta score of 15 or more, signs of venous claudication, recurrent upper leg and groin varicosities and/or venous ulcerations. Inclusion and exclusion criteria are listed in Table 1.

**Table 1:** Inclusion and exclusion criteria for this study

Inclusion	Exclusion
- Age between 18–65 years - Objectively documented CVD - Duplex ultra-sound suspected chronic deep vein obstruction (no DVT) - Patient scheduled for CE-MRV - Patient able to undergo CE-MRV twice within 2 weeks - Patient not scheduled to receive any treatment between CE-MRV examinations	- Hemodynamic instability - Known allergy for gadolinium-based MRI contrast agents - eGFR: < 30 mL/min 1.73 m^2^ - Claustrophobia - Pregnancy

CVD: chronic venous disease. DVT: deep vein thrombosis. CE-MRV: contrast-enhanced magnetic resonance venography. MRI: magnetic resonance imaging. eGFR: estimated glomerular filtration rate.

The study protocol required patients to be scanned twice, within a 2-week interval. A minimum of 3 days between the two scans was required to ensure no residual enhancement of the previously administered contrast agent [12, 15].

21/129 individuals (16.3%) gave written informed consent; one patient did not undergo the entire protocol for logistical reasons. Hence, 20 patients (11 men, 9 women; mean age 54, SD 11.8 years; range 36–77 years, BMI 23.6 + 2.5) were enrolled. The study protocol was approved by the local ethics committee.

### Magnetic resonance imaging (MRI) protocols

All MR examinations were performed on a 1.5-T MRI system (Intera, Philips Healthcare, Best, The Netherlands). For signal reception, a dedicated 12-element phased-array peripheral vascular coil with a cranio-caudal coverage of 128 cm (Philips) was used. Patients were imaged in a supine position.

Prior to contrast delivery, all patients underwent a standard 2D non-contrast-enhanced balanced turbo field echo (BTFE) sequence to visualize the abdominal and pelvic veins. This was followed by contrast material injection which was administered intravenously at 1.0 mL/s in the median cubital vein followed by 20 mL of saline flush injected at the same flow rate, using a remote-controlled dual-head injector (Spectris; Bayer Medrad, Indianola, PA, USA). Acquisition of the first scan volume was started 30 seconds after contrast administration.

A 3D ultra-fast gradient echo sequence (Ultrafast GE, THRIVE, Philips Healthcare) with fat suppression (spectral pre-saturation with inversion recovery, SPIR) was used for high-resolution steady-state imaging of the venous vasculature, ensuring coverage of at least the popliteal veins up to the suprarenal inferior caval vein. Like the first examination, the second examination consisted of the sequences mentioned above with addition of the steady-state gradient echo sequence (HR-FFE) without fat suppression. For both examinations, the order of the scanned sequences is listed in Table 2.

**Table 2:** Order of sequences for each examination

Examination I	Examination II
BTFE sequence	BTFE sequence
Contrast administration (gadobutrol)	Contrast administration (gadofosveset-trisodium)
Ultrafast spoiled GE	Ultrafast spoiled GE
	HR-FFE

For the first examination, a standard extracellular gadolinium agent gadobutrol (Gadovist, Bayer Schering Pharma, Berlin, Germany, now: Gadavist, Bayer HealthCare, Berlin, Germany) was administered. To mimic the steady-state of the high-relaxivity agent gadofosveset-trisodium, we used a double dose (2x) of the regular gadolinium-based agent (0.2 mL per kg body weight, equals 0.2 mmol/kg) [13–16].

For the second examination at 7 + 3 days, the blood pool contrast agent gadofosveset-trisodium was used (Ablavar, Lantheus Medical Imaging, Billerica, MA, USA). All patients received a fixed dose of 10 mL of gadofosveset-trisodium (0.25 mmol/mL). An overview of the detailed scan parameters is provided in Table 3.

**Table 3:** Scan parameters of the sequences used

	BTFE Abdomen / pelvis	Ultrafast spoiled GE	HR FFE Legs	HR FFE abdomen / pelvis
Scan mode	M2D	3D	3D	3D
Repetition time (TR) (ms)	3.8	7.8	12	12
Echo time (TE) (ms)	1.92	3.90	1.91	1.70
Flip angle (degrees)	65	10	20	20
Acquisition time (TA) (min) (for all stations)	6:40	14:52	13:37	7:48
Bandwidth (BW) (Hz)	595	181.8	159.4	186
Acquisition voxel (mm)	1.19 × 1.40 × 6.00	0.95 × 0.95 × 3.00	0.84 × 0.84 × 1.00	0.98 × 0.98 × 2.00
Reconstructed voxel (mm)	1.04 × 1.04 × 6.00	0.95 × 0.95 × 1.50	0.84 × 0.84 × 1.00	0.98 × 0.98 × 1.00
Number of slices	100	150 × 5 (750)	175 × 3 (525)	200 × 2 (400)
Acquisition matrix	336 × 228	380 × 266	560 × 392	560 × 392
FoV	400 × 319	400 × 280	470 × 329	470 × 329
Fat Supression	No	SPIR	No	No
Cardiac synchronisation (ECG)	Yes	No	No	No

The BTFE sequence was acquired in two volumes to cover the abdomen and pelvis. The ultra-fast GE sequence was acquired using a coronal acquisition scheme in three volumes which were stitched and then reconstructed in the axial plane on the scanner. The HR-FFE was also acquired in three coronal volumes. Stitching is not available for this sequence, for each volume axial reconstructions were made on the scanner. The acquired volume for the 3D scans covered the deep vein system from the inferior vena cava (IVC) to the distal popliteal vein. The calf veins are not routinely included in our scan protocol for two reasons. First, inter-individual patient length varies (on average from 1.40 meters to 2.00 meters) which results in variable coverage of the calves. Second, findings in the (proximal) calf veins do not have consequences for treatment.

## Evaluation of studies

**Fig. 1:**
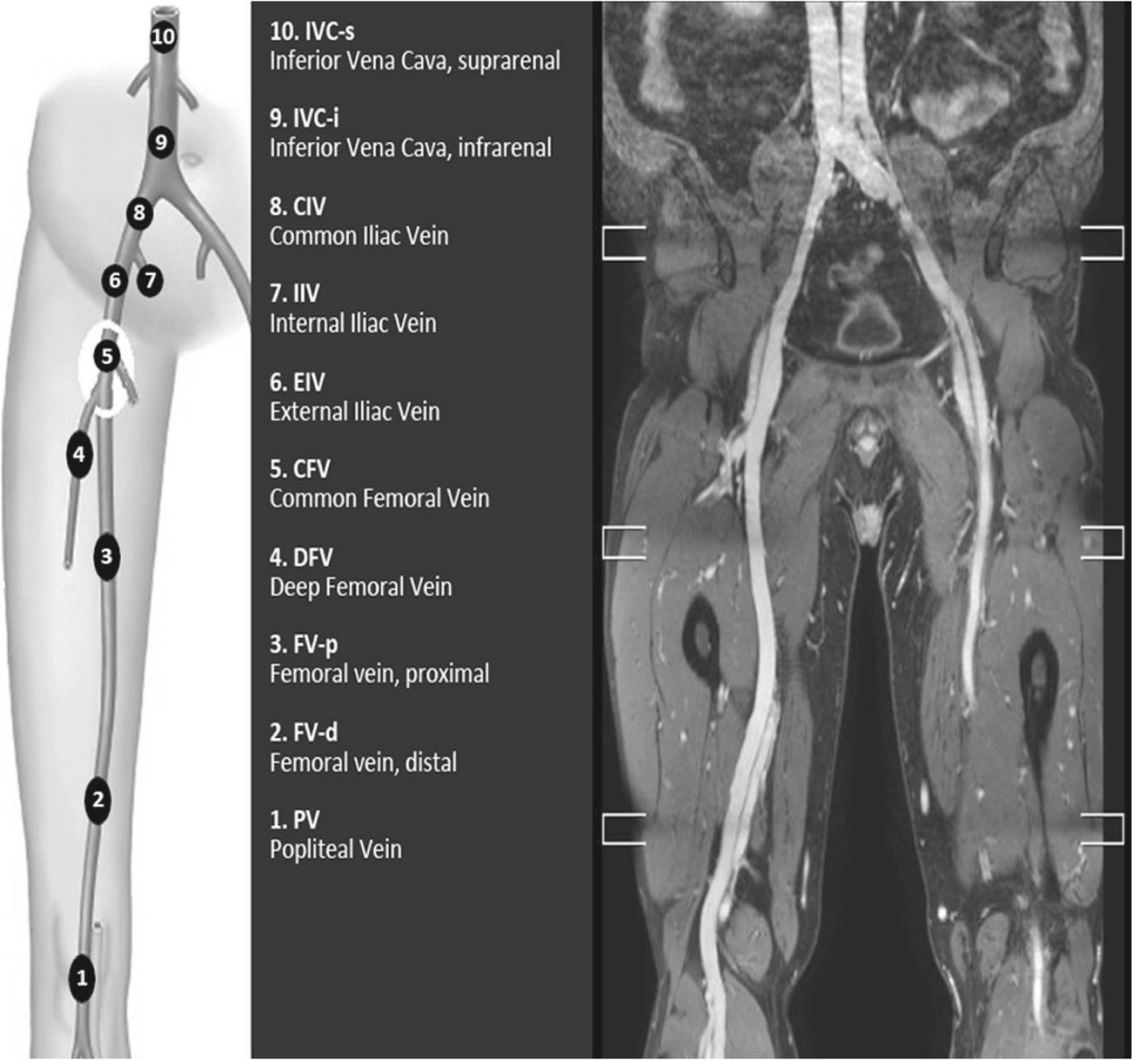
Schematic and MR venography overview of the evaluated vein segments

All sequences were evaluated by two independent reviewers: 1 (CWKP) and 2 (NI), both blinded for the contrast material used, individual scan dates and each other’s results. Reviewer 1 had 5 years of experience with venous vascular MR studies specifically, and reviewer 2 had 1 year of experience. Each sequence was evaluated separately within different sessions. Both reviewers had access to the source images as well as common post-processing tools [multi-planar reconstruction (MPR)/curved planar reconstruction, maximum intensity projection (MIP)]. The reviewers were blinded for the clinical record of the patients. The following vessel segments were evaluated: 1: popliteal vein, 2: distal femoral vein, 3: proximal femoral vein, 4: profunda femoral vein, 5: common femoral vein, 6: external iliac vein, 7: internal iliac vein, 8: common iliac vein, 9: infrarenal inferior caval vein, 10: suprarenal inferior caval vein (Fig. 1).

In all patients, both legs were evaluated, allowing for evaluation of 360 vessel segments in total.

**Fig. 2:**
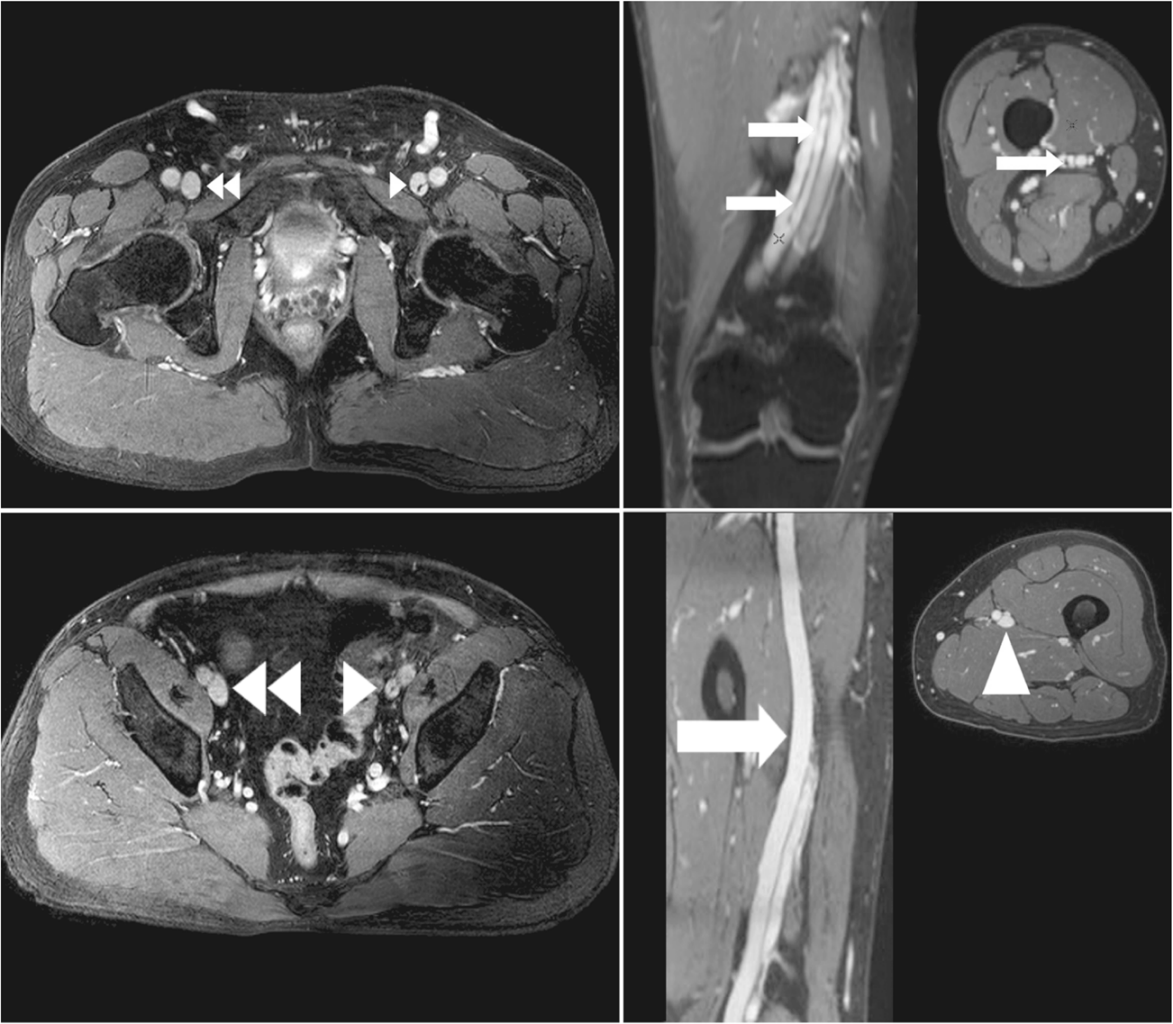
Example of post-thrombotic changes as visualised with MR venography. Top left: normal right common iliac vein (*double arrowhead*). Black strands in left common iliac vein (*arrowhead*) which are residual scarring/trabeculations after deep vein thrombosis. Bottom left: normal right external iliac vein. Similar scarring is seen in the left external iliac vein compared to the common femoral vein with the addition of >50% luminal narrowing compared to the right. Top right: coronal reconstruction showing scarring (*arrows*) of the femoral vein. Bottom right: coronal reconstruction showing a normal femoral vein without any (post-thrombotic) scarring (*large arrow*, *large arrowhead*)

The following items were subjectively scored: image quality, confidence of image interpretation and findings. Image quality was scored on a Likert-like scale from 1 to 5, with 1: not visualised, 2: poor, 3: fair, 4: good and 5: excellent. Image confidence was scored on a scale from 1 to 4, with 1: unsure, 2: mildly confident, 3: moderately confident and 4 very confident. Scoring systems used have been outlined before [10, 17, 18]. The image findings analysed were those associated with post-thrombotic obstructive disease: post-thrombotic scarring or trabeculations with or without severe luminal narrowing. On MRV, these scars or trabeculations are visible as hypo-intense dots or strands with or without a decreased size of the diseased vein (compared to, for example, a not diseased contralateral vein). Examples are shown in Fig. 2.

If present, artefacts caused by parallel imaging reconstruction such as aliasing and ringing were registered.

Confidence of image interpretation was scored on a scale from 1 to 4, with 1: unsure, 2: mildly confident, 3: moderately confident and 4: confident. Image findings were scored as either 0: no post-thrombotic changes or 1: post-thrombotic changes. Post-thrombotic changes were defined as visible post-thrombotic remnants such as vein scarring, lumen obstruction and/or collateral formation [6]. Left and right leg vessel segments were grouped for qualitative analysis.

Finally, reviewer 1 measured vein and muscle signal intensity (S) for each vessel segment using the single acquisition technique for quantitative analysis described by Firbank et al. [19]. Background noise was determined by placing a 500-pixel region of interest (ROI) in an artefact-free area of air. All measurements were performed at the level of the venous ROIs which were placed in the centre of the vessel segment. Noise values were corrected for magnitude effects by the Rayleigh factor of 0.665 [20]. The signal-to-noise ratio (SNR) was calculated by SNR = 0.655∙S/σ, with σ being the standard deviation of the signal in air. The CNR for the vessel segments was calculated as follows:

$$ {\mathrm{CNR}}_{\mathrm{vein}}=\left({\mathrm{SNR}}_{\mathrm{vein}} - {\mathrm{SNR}}_{\mathrm{muscle}}\right) $$

## Statistical analysis

To evaluate the degree of agreement among the two reviewers, the kappa value was calculated for image quality, image interpretation and image findings. Cohen’s kappa coefficients of agreement between observers were determined for each feature. Agreement was based on the Fleiss classification: <0.40, poor; 0.40–0.59, moderate; 0.60–0.75, good; >0.75, excellent) [21].

Generalized estimating equations (GEEs) were used to assess the effect of the imaging techniques and contrast material on detection of intravenous disease changes, excellent image quality (score of 5) and very confident interpretation (score of 4). The reason to use GEEs with the logit link function was to correct for repeated measurements within the same patients (same patients and segments, different techniques). Additionally, we corrected for metallic implants.

A *p* value < 0.05 was considered statistically significant. All calculations were performed using Microsoft Excel 2013 (Microsoft Office; Microsoft, Redmond, WA, USA) and IBM SPSS Statistics for Windows version 23.0 (IBM Corp. Armonk, NY, USA).

## Results

Inter-observer agreement with regard to image quality was excellent between all three sequences with a kappa of 0.95. Inter-observer agreement with regard to confidence of image interpretation and image findings were excellent as well with a kappa of 0.85 and 0.84, respectively.

### Image quality

Comparison of image quality between both ultra-fast GE sequences and the HR-FFE sequence showed an overall high image quality for all sequences (91.5%, excellent score; Table 4).

**Table 4:** Average scores of image quality per segment

	Sequence		
Vein segment	Ultrafast GE (gadobutrol)	Ultrafast GE (gadofosveset-trisodium)	HR FFE
v. poplitea	3.75	3.8	3.8
v. fem. Dist.	3.85	4	3.8
v. fem. Prox.	3.95	3.9	3.8
v. profunda fem.	4	3.95	3.8
v. fem. Com.	4	3.95	3.8
v. iliaca ext.	3.9	3.85	3.9
v. iliaca int.	4	3.95	3.85
v. iliaca com.	3.95	3.9	3.75
Inferior caval vein (infrarenal)	4	3.95	3.1
Inferior caval vein (suprarenal)	3,95	3,95	3
Average of all segments	3,94 (+0,35)	3,92 (+0,31)	3,7 (+0,82)

**Fig. 3:**
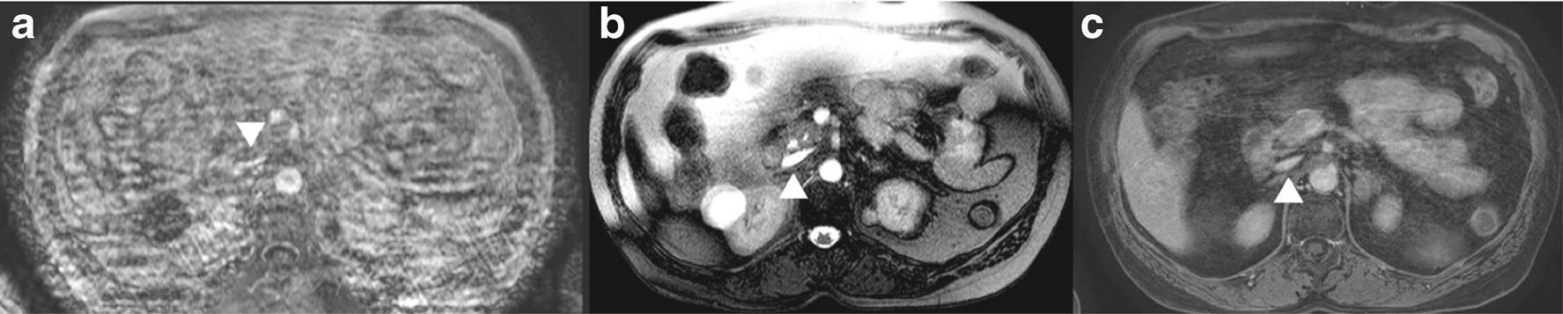
Example of inferior vena cava image quality. **A**) HR-FFE (gadofosveset-trisodium), **B**) BTFE (non-contrast-enhanced), **C**) Ultra-fast GE (gadofosveset-trisodium) sequence. All images show the inferior vena cava (supra-renal) at the same level in the same patient during the same examination (*arrowhead*). The apparent motion artifacts distort the image of the inferior vena cava only on the HR-FFE image

Overall, there was a significant difference between the three groups (*p* = 0.045) in favour of the ultra-fast GE sequences. In particular, the image quality of the ultra-fast GE sequence with gadobutrol showed more often an excellent reported quality in comparison to the HR-FFE sequence (*p* = 0.013) (Fig. 3).

There were no statistically significant differences (p = 0.578) in the reported image quality for the ultra-fast GE images from examination 1 (gadobutrol) compared to the ultra-fast GE images from examination 2 (gadofosveset-trisodium).

### Image interpretation

The reported confidence of interpretation was high for all three imaging techniques (95.5%, very confident). Overall (*p* = 0.139), as well as between the techniques (*p* = 0.295) and the two contrast materials administrated (*p* = 0.670), there was no statistically significant difference in confidence of interpretation.

**Fig. 4:**
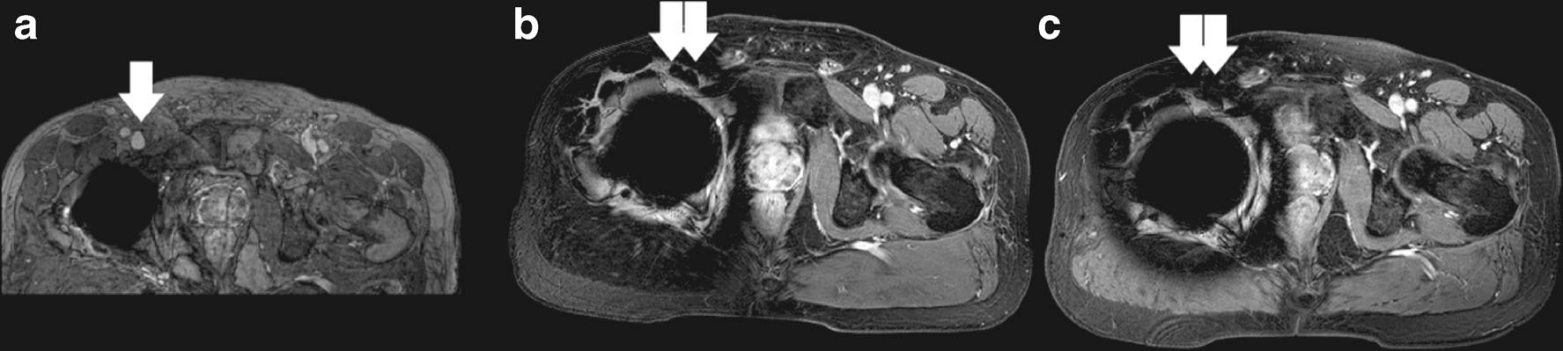
Metal artifacts caused by hip implant. **A**) HR-FFE sequence (gadofosveset-trisodium), limited artifacts with still a visible common femoral vein (*arrow*). **B**) Ultra-fast GE (gadofosveset-trisodium) sequence with severe artifacts (*double arrow*) and unsure interpretation of the vascular structures. **C**) Ultra-fast GE (gadobutrol) sequence with the same severe artifacts (*double arrow*) as in **B**)

In three patients, a lower confidence of interpretation was noted at the level of a metallic joint or spinal implant specifically on the ultra-fast GE sequences, which, in comparison, did not affect confidence of interpretation on the HR-FFE sequence (Fig. 4).

### Image findings

**Fig. 5:**
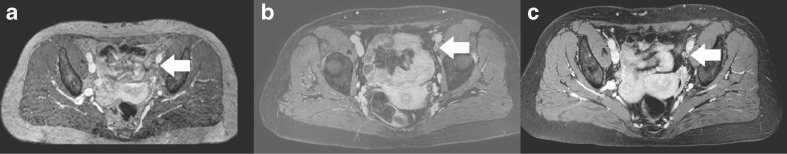
Axial reconstructions in a patient with chronic obstruction of the external iliac vein. **A**) HR-FFE (gadofosveset-trisodium) sequence showing the typical appearance of an obstructed and shrivelled external iliac vein with trabeculae (*arrow*) as a sign of post-thrombotic changes. **B**) Appearance of the external iliac vein on the ultra-fast GE (gadofosveset-trisodium) sequence. **C**) Appearance of the external iliac vein on the ultra-fast GE (gadobutrol) sequence

**Fig. 6:**
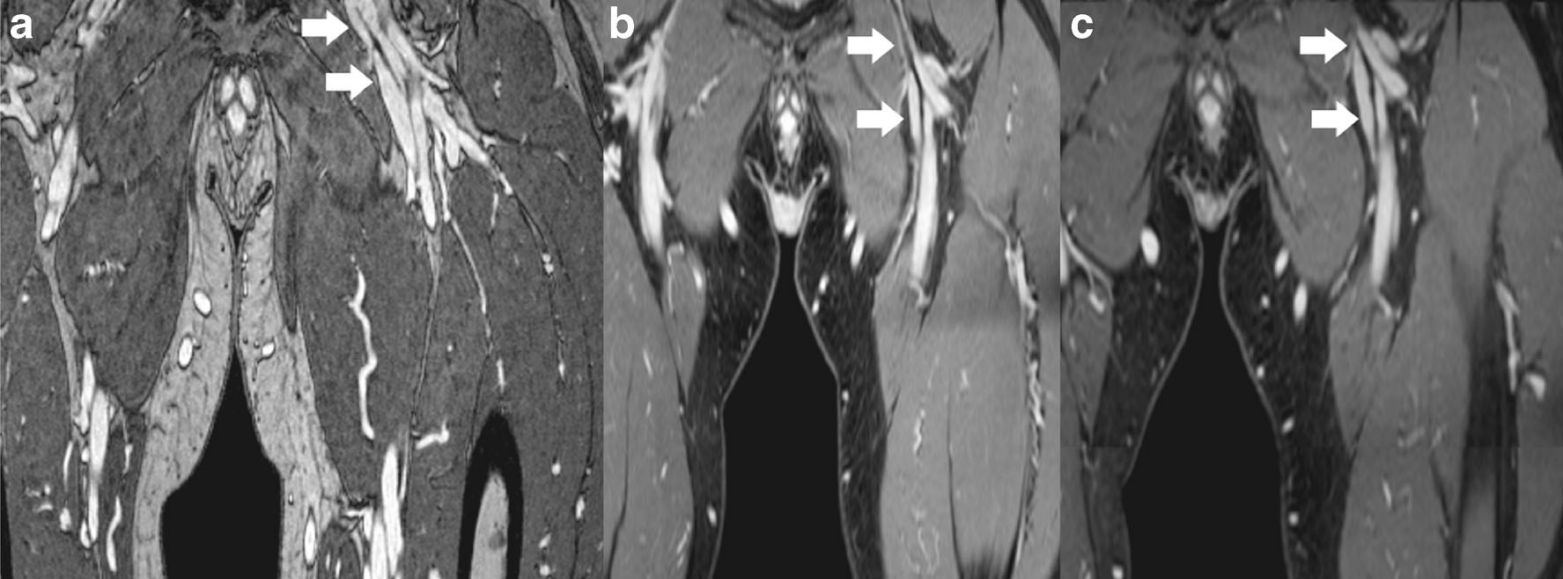
Coronal reconstructions of an obstructed external iliac and common femoral vein. **A**) HR-FFE (gadofosveset-trisodium) sequence shows fibrotic strands in the external iliac and common femoral vein (*arrows*). **B**) Ultra-fast GE (gadofosveset-trisodium) sequence and **C**) Ultra-fast GE (gadobutrol) sequence of the same vein segments as **A**), showing the same post-thrombotic changes (*arrows*)

There was a high consistency with regard to image findings between the different scan sequences and contrast materials used. Examples are shown in Figs. 5 and 6.

GEEs yielded no significant differences between all groups in regard to image findings (*p* = 0.328). More specifically, no significant differences were observed between HR-FFE gadofosveset-trisodium vs. ultra-fast GE gadobutrol (*p* = 0.547) and ultra-fast GE gadofosveset-trisodium vs. ultra-fast GE gadobutrol (*p* = 0.527).

### CNR

CNR ratios for the contrast-enhanced sequences were comparable for both contrast material and imaging techniques, as shown in Table 5.

**Table 5:** Contrast-to-noise ratio measured for each vessel segment per sequence

Venous segment	Ultrafast GE MRV (Exam I)	Ultrafast GE MRV (exam II)	HR SteadyState MRV (exam II)
1 V. Pop	52 (+15)	73 (+21)	29 (+24)
2 V fem. dist.	44 (+22)	66 (+37)	47 (+28)
3 V. fem. prox.	58 (+18)	50 (+8)	37 (+16)
4 V. prof. fem.	42 (+18)	51 (+6)	37 (+22)
5 V. fem. com.	29 (+22)	24 (+6)	41 (+26)
6 V ext. iliac	37 (+30)	34 (+16)	30 (+30)
7 V. int. iliac	43 (+44)	36 (+30)	55 (+20)
8 V. com. iliac	43 (+30)	41 (+42)	52 (+24)
9 IVC infra	34 (+24)	35 (+24)	40 (+38)
10 IVC supra	58 (+33)	43 (+35)	42 (+34)
Average for all segments	44 (+25)	45(+23)	41(+26)

Data listed as mean (+ SD)

## Discussion

Performing high-resolution MRV with a regular gadolinium-based agent such as gadobutrol instead of a blood pool contrast agent such as gadofosveset-trisodium is possible, allowing for high-quality MRV studies. Even though the two contrast agents used are different in terms of concentration and protein binding, we did not find any significant differences in reported image quality, confidence of interpretation or image findings.

In our daily practice, we used gadofosveset-trisodium as contrast material of choice for MRV. With regard to contrast clearance after injection, 94% of gadofosveset-trisodium is cleared after 72 hours compared to gadobutrol that is cleared 90% after 12 hours [22–24]. To prevent any interference related to the prolonged clearance time of the blood pool agent, the initial scan of the study protocol was performed using gadobutrol and a safety margin of 3 days was used to allow for (near) complete clearance of the contrast administered. Since patients with chronic venous disease generally have stable disease no confounding factor was introduced by allowing 3 to a maximum of 14 days in between the two scans [25].

Secondly, the reported findings with the ultra-fast GE sequence in comparison to the HR-FFE sequence are virtually equal. Additionally, we observed a slight increase in overall image quality using the ultra-fast GE sequence. This particularly holds true for the abdomino-pelvic segments, which are regarded as the most important segments in clinical practice [6]. The main reason for the acquisition of the non-contrast enhanced BTFE images in our study protocol were well known evaluation problems of the inferior vena cava on the HR-FFE sequence. In 6 out of 20 patients we observed image quality problems due to motion artefacts which hampered assessment on the HR-FFE sequence, that were not encountered on the ultra-fast GE sequence. This implies an additional benefit in terms of confidence and reduction in scan time (non-contrast enhanced acquisitions can be omitted) when implementing an ultra-fast GE sequence to the scan protocol.

Using the ultra-fast GE-sequence instead of the HR-FFE + BTFE sequences implies a nearly 50% reduction in acquisition time from 28 min to 15 minutes.

There still are some patients that will benefit from HR-FFE scanning. In patients with metallic prosthesis of the hip, knee or spine the image quality of the ultra-fast GE sequence can be mediocre. In 3 segments we encountered more severe artefacts on the ultra-fast GE sequences in comparison to the HR-FFE sequence related to hipjoint and spinal implants. This did affect confidence of interpretation but did not result in general impairment of the results reported for these MRV studies. In our practice we have not encountered MR acquisition issues with inferior vena cava filters, unfortunately none were present in the studied patients to compare image quality for these specific implants.

With regard to the contrast material used we did not find any significant difference in reported image quality, confidence of interpretation or image findings. We have shown that performing MRV with a regular gadolinium based agent such as gadobutrol is feasible, allowing for high quality MRV studies all over the world. Interesting to note is that comparing gadobutrol to gadofosveset-trisodium the confidence interval shows gadobutrol being potentially slightly better for detection of disease.

## Limitations of this study

Since there are no previous studies investigating the possibility of replacing a high relaxivity agent with a regular gadolinium based contrast agent for MRV specifically, we had to set up our protocol based on research for MR-angiography in other vascular territories. We had to assume that double dose of the regular gadolinium based contrast agent gadobutrol provided enough relaxivity to ‘mimic’ gadofosveset-trisodium even though the reported relaxivity for a single dose of gadobutrol is 5.5 compared to 19 for gadofosveset-trisodium. Fortunately, considering the current unavailability of a high relaxivity agent with a vascular indication in Europe, our study results show that a regular gadolinium-based agent can be an alternative for MRV. Furthermore we could not randomize the order of the administration of the contrast agents within our study design. To ensure no interference due to the prolonged clearance of gadofosveset-trisodium (more than 2 weeks) gadobutrol was always given first. However we acknowledge that a cross-over design would have been more ideal. The 20 patients included provided us with 3 x 18 measurements (=54) per patient. The calculated intra-class correlation (ICC) for identification of a diseased segment was 0.325, which is relatively high. This means that the repeated measurements show some correlation. This implies that statistically we cannot interpret all measurements as completely independent. Considering the amount of measurements (20 x 54 = 1080) the study size is still adequate for our statistical analysis and the conclusions of our study.

## Conclusions

For high-resolution MRV, it is possible to use a regular gadolinium-based agent (gadobutrol) instead of the blood pool agent gadofosveset-trisodium. Furthermore, using an ultra-fast GE sequence for MRV can considerably shorten the scan time for the majority of patients without loss of image quality or diagnostic yield.

## Abbreviations

MRI: Magnetic resonance imaging
MRV: Magnetic resonance venography
GRE: Gradient echo
GE: Gradient echo
HR-FFE: High-resolution fast field echo
CTV: Computed tomography venography
NIVL: Non-thrombotic iliac vein lesion
DUS: Duplex ultrasound
BTFE: Balanced turbo field echo
SPIR: Spectral pre-saturation with inversion recovery
ROI: Region of interest
CNR: Contrast to noise ratio
MPR: Multi-planar reconstruction
MIP: Maximum intensity projection
IVC: Inferior vena cava
